# Supplementary material for: Challenging the Astral mass analyzer to quantify up to 5,300 proteins per single cell at unseen accuracy to uncover cellular heterogeneity
Source: Nat Methods. 2025 Jan 16;22(3):510–9. doi: 10.1038/s41592-024-02559-1 (PMC11903296; doi:10.1038/s41592-024-02559-1)
Supplement: Supplementary file 1 — Supplementary Notes, Table 1 and Figs. 1–15. [file 41592_2024_2559_MOESM1_ESM.pdf]

nature methods

Supplementary information

https://doi.org/10.1038/s41592-024-02559-1

# Challenging the Astral mass analyzer to quantify up to 5,300 proteins per single cell at unseen accuracy to uncover cellular heterogeneity

In the format provided by the authors and unedited

# Contents


**Supplementary Notes** **2**

Benchmarking label-free single-cell sample preparation . . . 2

**List of Supplementary Tables**

S1 LC gradient details. . . . 3

**List of Supplementary Figures**

S1 Influence of search strategy on the total number of identified precursors, protein groups, long-term reproducibility and FDR. . . . 4

S2 Degree of potential carryover dependent on gradient length. . . . 5

S3 Dilution series – from 10 ng to 50 pg sub-single cell level . . . 6

S4 Base-peak chromatograms with and without FAIMS Pro interface. . . . 7

S5 Peptides intersection and peptides abundances in runs with and without FAIMS Pro interface. . . . 7

S6 Human–Yeast proteome mix to assess quantitative precision and accuracy of Orbitrap Astral and Orbitrap Exploris 480 mass spectrometers using Ms1-based quantitation with minimum 2 peptides . 8

S7 Human–Yeast proteome mix to assess quantitative precision and accuracy of Orbitrap Astral and Orbitrap Exploris 480 mass spectrometers. MS2-based quantitation. . . . 9

S8 Workflow benchmark. One-Pot 384-well in comparison with LF 48 and EVO 96 ProteoCHIPs. . . . 10

S9 Data completeness and Coefficients of Variation in A549 dataset. . . . 11

S10 Data completeness and Coefficients of Variation in TE/hPSC dataset. . . . 12

S11 GO analysis of upregulated proteins in TE-like cells with highlighted selected biological pathways (single-cell data). . . . 13

S12 Distribution of protein abundance in TE vs hPSC cells on a PCA plot (100-cells data). . . . 14

S13 GO analysis of upregulated proteins in TE cells with highlighted selected biological pathways (100-cells data). . . . 15

S14 Gating strategy for the isolation of individual TE cells using flow cytometry. . . . 16

S15 Gating strategy for the isolation of individual hPSCs using flow cytometry . . . 17

# Supplementary Notes

## Benchmarking label-free single-cell sample preparation

To decide on an optimal label-free sample preparation strategy using the cellenONE robot, we benchmarked some protocols established on that machine. We decided to use the One-Pot 384-well sample preparation published earlier in our group[1] as a reference point and compared it to the commercial proteoCHIP LF 48[2] as well as to the more recently commercialized proteoCHIP EVO 96 protocol of CellenION.[3] While the One-Pot 384-well workflow has a working volume of 1 µL this can be reduced to 300 nL in the proteoCHIP workflows enabling a higher sample and protease concentration that we expect to reduce losses and reduce excess trypsin needed. As a tradeoff, a direct injection from the proteoCHIP to the LC-MS system is tricky and bears the risk of injecting hexadecane into the MS system (in case of the LF 48 workflow, no hexadecane was used in the EVO 96 chip). This requires a sample transfer step that is done either by manual pipetting (LF 48) or by centrifugation (EVO 96) to a 96-well low-binding PCR plate. For each workflow blanks were processed in the same plate or chip and contained all reagents and buffers but no cell.

In our first attempt, the re-used and mildly washed proteoCHIP LF 48 by far outperformed the One-Pot 384-well workflow by means of 68% more PGs identified. However, out of 1971 (PGs) identified using the LF 48 chip, 685 (34%) were identified in a blank control, that contained all reagents and received the same treatment as single-cell samples, but no cell was added (Supplemental Figure 8 a). This significant background contamination very likely results from previous runs performed in the (re-used) chip. To reduce the background, we implemented a stringent washing protocol for the Teflon LF 48 chip. As a result, the background level of our blank control was reduced to the same level as seen for 384-well plates but the ID numbers were reduced to 1087 PGs, slightly less than what we obtained using the 384-well plate (1174 PGs). We hypothesize that contamination peptides covered the (non-stringent cleaned) surface of the (multiple used) LF 48 chips serving as a carrier, but potentially covering real (regulated) proteins.

Next, we compared the more recent (multiple used and washed) EVO 96 chip. Both, the EVO 96 chip and the well surface of our 384-well plate are made of polypropylene. Both workflows were performed from the same batch of cells on the same day and using a very similar lysis and digestion solution. In conclusion, the main difference between both workflows is a: the volume during digestion (1 µLand 300 nL for 384-well and EVO 96 chip respectively) and b: the final sample transfer step to a 96-well PCR plate done only for the EVO 96 workflow. The One-Pot 384-well protocol thereby yielded more identified PGs even at lower background. Selecting only big (> 20 µm) A549 cells yielded 3185 PGs on average using the One-Pot 384-well workflow, compared to 2220 PGs using the EVO 96 chip. Based on our previously published results[**Matzinger2022**] showing the effect of sample transfer, we hypothesize this being the main reason for a slightly lowered performance of the EVO 96 workflow. (Supplemental Figure 3 b).

We conclude that, in our hands, the One-Pot 384-well workflow is the best suited for sample preparation due to competitive or even superior ID numbers and simpler handling as no transfer step or removal of hexadecane is needed. Furthermore, reporting blanks seems of high importance to us to check on background contamination, which is especially problematic in case of economic multiple usage of chip materials. All single-cell samples in this study were therefore prepared using the One-Pot 384-well[**Matzinger2022**] workflow.

# Supplementary Tables

| | 30 SPD | | 40 SPD | | 50 SPD | | 60 SPD | | 80 SPD | |
|---|---|---|---|---|---|---|---|---|---|---|
| time, min | flow, nL/min | %B | flow, nL/min | %B | flow, nL/min | %B | flow, nL/min | %B | flow, nL/min | %B |
| 0 | 250 | 5 | 450 | 5 | 450 | 1 | 450 | 1 | 500 | 4 |
| 0.1 | - | - | - | - | 450 | 4 | 450 | 4 | 500 | 4 |
| 0.8 | 250 | 8 | 450 | 8 | - | - | - | - | - | - |
| 1.0 | - | - | 450 | 10 | - | - | - | - | - | - |
| 1.1 | - | - | 250 | 10.3 | - | - | - | - | - | - |
| 1.6 | - | - | - | - | - | - | - | - | 500 | 12 |
| 1.7 | - | - | - | - | - | - | - | - | 200 | - |
| 1.9 | - | - | - | - | 450 | 12 | 450 | 12 | - | - |
| 2 | - | - | - | - | 200 | - | 200 | - | - | - |
| 9.7 | - | - | - | - | - | - | - | - | 200 | 28.5 |
| 11.2 | - | - | - | - | - | - | - | - | 200 | 40 |
| 12 | - | - | - | - | 200 | 22.5 | - | - | 300 | 99 |
| 13.5 | - | - | - | - | - | - | 200 | 28.5 | - | - |
| 14 | - | - | - | - | - | - | - | - | 500 | 99 |
| 17 | - | - | - | - | - | - | 200 | 40 | - | - |
| 18 | - | - | - | - | - | - | 300 | 99 | - | - |
| 19.5 | - | - | - | - | 200 | 40 | - | - | - | - |
| 20 | - | - | - | - | - | - | 300 | 99 | - | - |
| 22 | - | - | - | - | 300 | 99 | - | - | - | - |
| 25 | - | - | - | - | 300 | 99 | - | - | - | - |
| 25.1 | - | - | 250 | 30 | - | - | - | - | - | - |
| 27.6 | - | - | 250 | 44 | - | - | - | - | - | - |
| 28.4 | - | - | 250 | 99 | - | - | - | - | - | - |
| 30.8 | 250 | 30 | - | - | - | - | - | - | - | - |
| 31.4 | - | - | 450 | 99 | - | - | - | - | - | - |
| 33.8 | 250 | 44 | - | - | - | - | - | - | - | - |
| 34.8 | 250 | 99 | - | - | - | - | - | - | - | - |
| 38.8 | 250 | 99 | - | - | - | - | - | - | - | - |

**Table S1: [LC gradient details.** LC gradient details over time for different SPD conditions. Buffer B: acetonitrile with 0.08% formic acid, buffer A: 0.1% formic acid

# Supplementary Figures

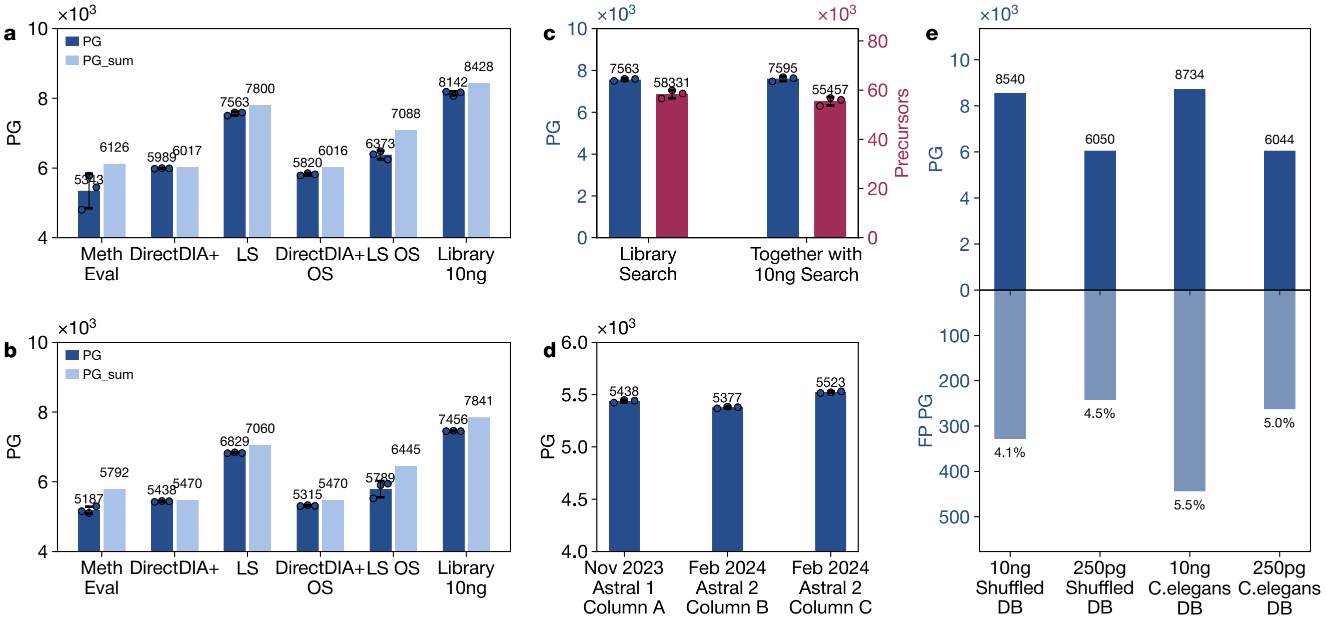


**Figure S1: Influence of search strategy on the total number of identified precursors, protein groups, long-term reproducibility and FDR.** 250 pg of HeLa (**a**, **c**, **e**), and K562 (**b**, **d**) peptides from diluted bulk digest were injected each. Circles indicate identified protein groups (PG) or precursors at 1% FDR in individual replicates, bars indicate their means, while error bars indicate standard deviations with n=3 technical replicates. In a & b, results shown in Figure 1 **d** & **h** are reflected for HeLa and K562 respectively, analyzed in the given mode using Spectronaut 18 at % FDR against a target (human proteome) and entrapment (shuffled human proteome and *C. elegans*) database. Bars indicate the total number of identified protein groups in the target database (top) and shuffled database (bottom) within n=3 technical replicates. In **c**, 250 pg HeLa was searched against a library created from 3 replicate measurements of 10 ng or co-searched together with them in DirectDIA+ mode. In **d**, 250 pg K562 was injected with the same settings as above mentioned and analyzed in DirectDIA+, comparing measurements from 2 batches (November 2023 and February 2024) on two different Orbitrap Astral MS and using 3 different columns.

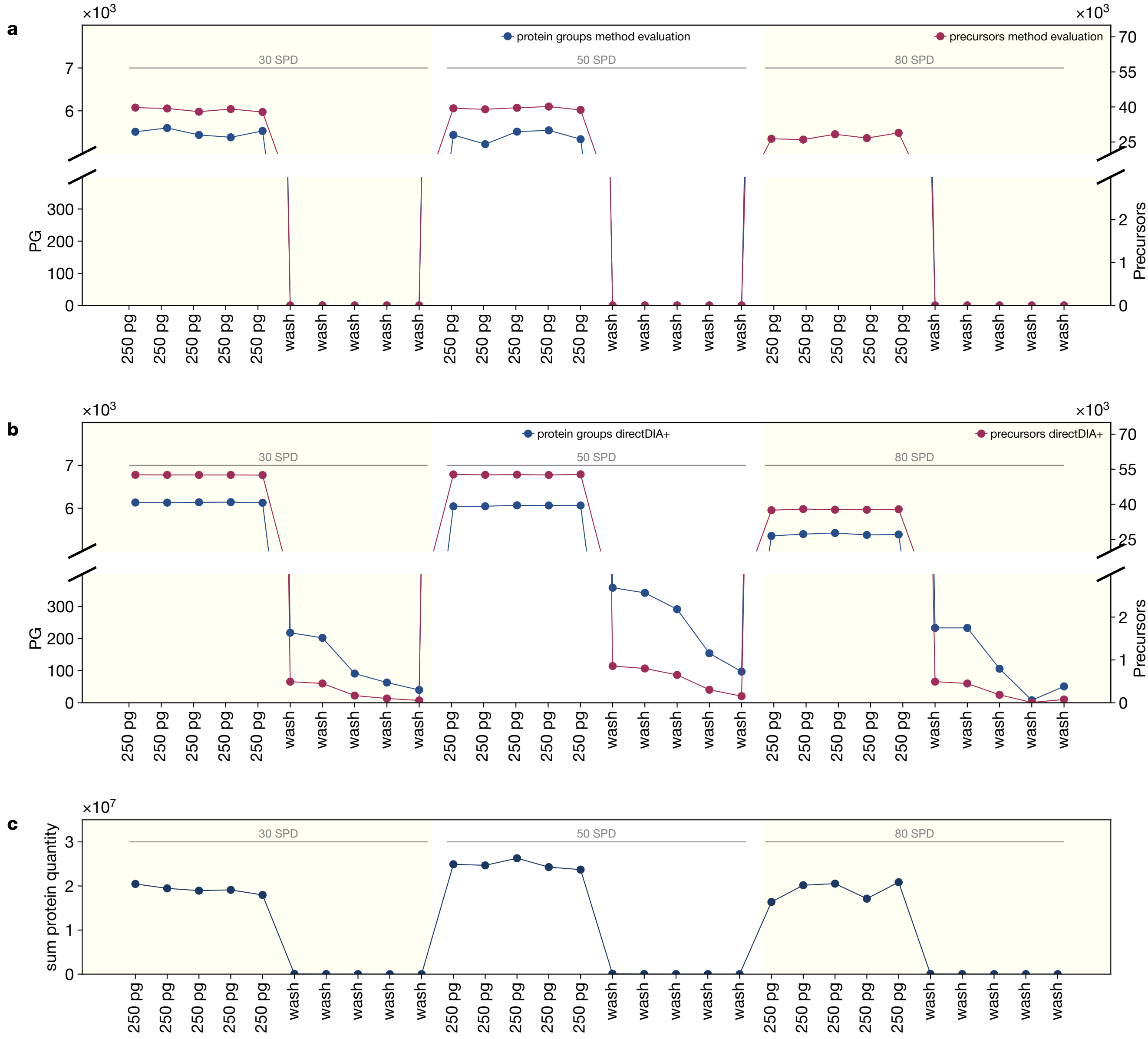


**Figure S2: Degree of potential carryover dependent on gradient length.** Identified protein groups and precursors from 250 pg HeLa diluted bulk and wash-runs, shown in the sequence as measured on the 25 cm IonOpticks column in direct injection mode and at a throughput as indicated. Data was analyzed in method evaluation mode without matching (**a**) or using directDIA+ (**b**) in Spectronaut 18. Panel **c** shows the non-normalized sum of all protein group quantities reported from the directDIA+ analysis.

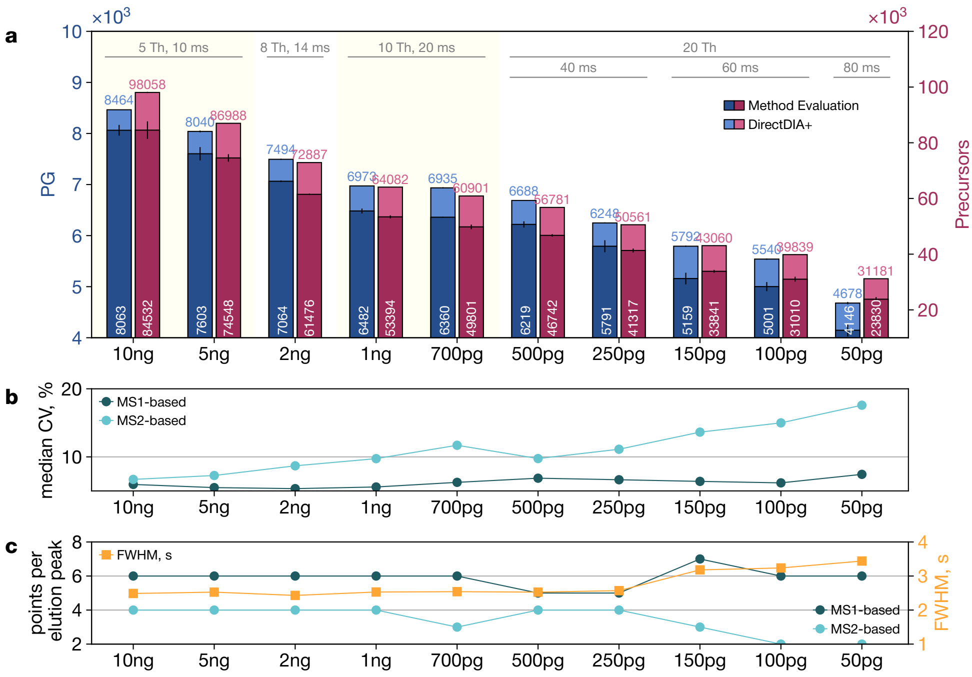


**Figure S3: Dilution series – from 10 ng to 50 pg sub-single cell level.** 50 pg – 10ng of HeLa peptides from the very same diluted bulk digest at 5ng/µL were injected each. Peptides were separated at 50 SPD throughput and data was recorded in DIA mode on the Orbitrap Astral MS and analyzed in direct DIA+ mode with or without usage of the method evaluation option as indicated using Spectronaut 18. In **a**, circles indicate identified PG or precursors at 1% FDR in individual replicates, bars indicate their means, while error bars indicate standard deviations with n=3. Dark colors represent identified proteins in method evaluation mode, with no matching across replicates and light colors indicate boosted ID numbers when allowing for matching. Acquiring parameter details are shown above. In **b**, dots indicate the obtained median CV on protein level for each gradient length (DirectDIA+) when performing quantification on MS1 or MS2 level respectively. In **c**, dots indicate the median number of data points for each precursor per elution peak that were used for quantification (DirectDIA+) for each gradient length on MS1 or MS2 level respectively as well as the median FWHM of elution peaks.

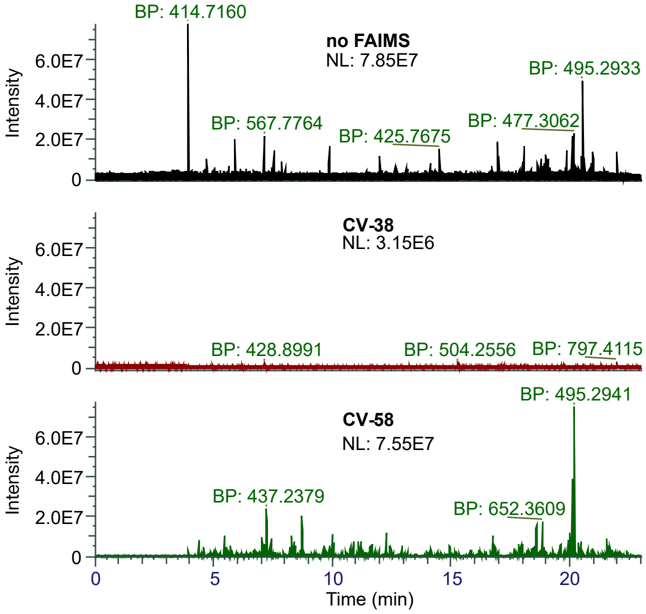


**Figure S4: Base-peak chromatograms with and without FAIMS Pro interface.** Base-peak chromatograms from representative replicate measurements from the data shown in Figure 4. 250 pg of HeLa peptides from diluted bulk digest were injected each. Peptides were separated at a throughput of 50 SPD. Data was recorded with or without the FAIMS unit attached and at the given compensation voltage.

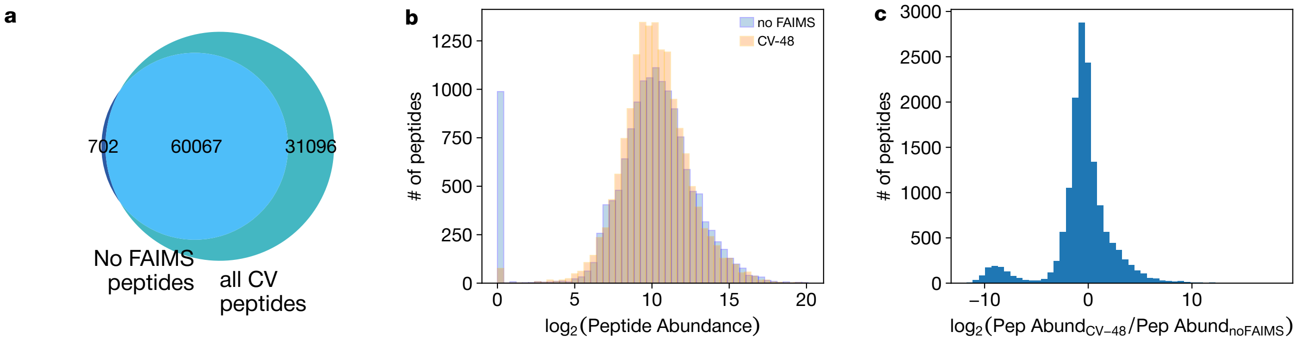


**Figure S5: Peptides intersection and peptides abundances in runs with and without FAIMS Pro interface.** The Venn diagram (**a**) shows the intersection of identified peptides in all acquired runs with and without FAIMS Pro interface. The Peptide abundance distribution (**b**) in runs with CV -48 V and no FAIMS interface runs and the distribution of $\log_2$-transformed peptide abundance ratios (**c**) of CV-48 vs noFAIMS runs is shown. Peptide abundances were calculated from MS1 level intensities, and all data of this Figure originates from **Figure 2** of the main study.

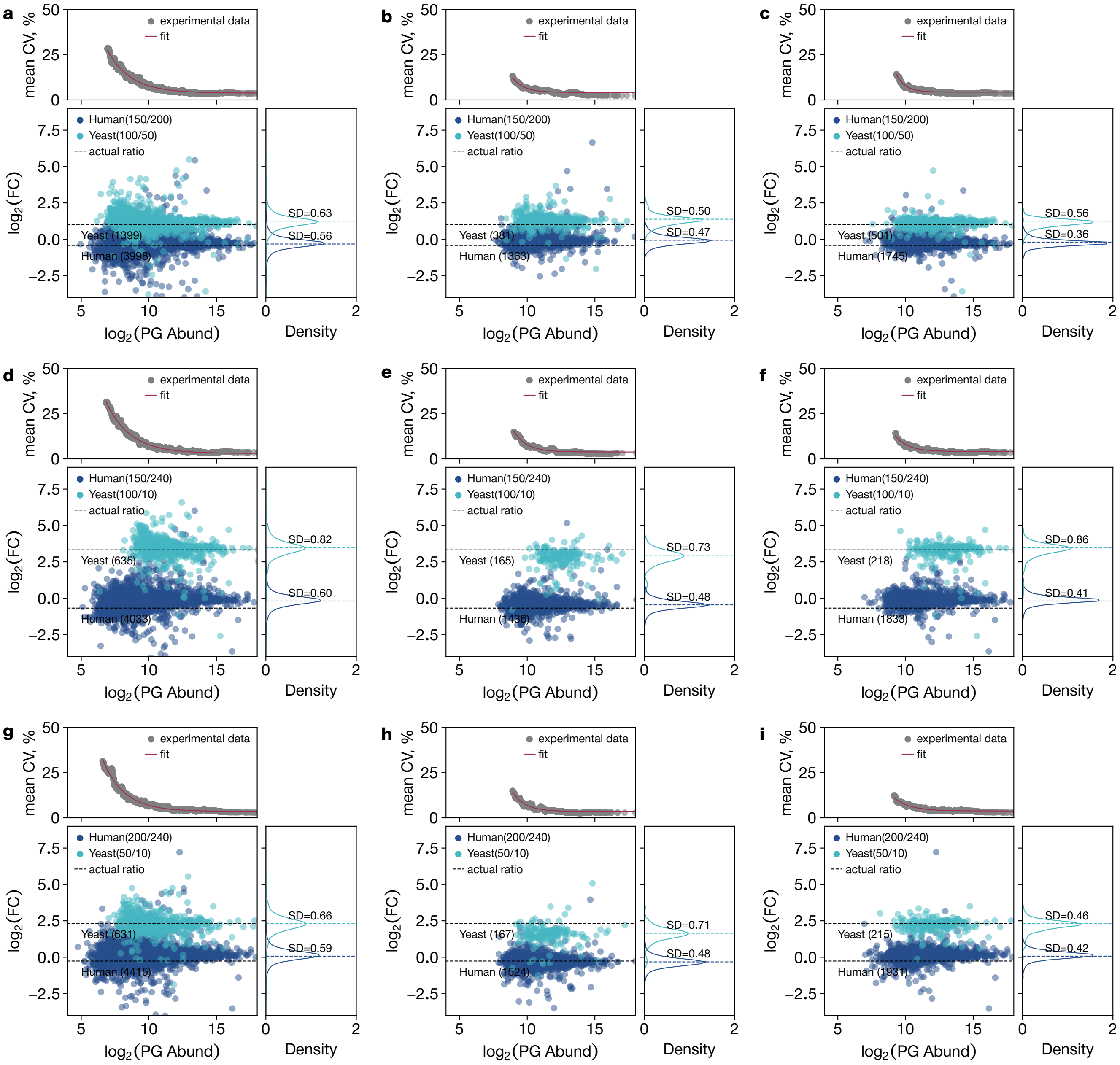


**Figure S6: Human–Yeast proteome mix to assess quantitative precision and accuracy of Orbitrap Astral and Orbitrap Exploris 480 mass spectrometers using Ms1-based quantitation with minimum 2 peptides.** From diluted bulk digests 250 pg two-proteome mixes consisting of 150 pg HeLa + 100 pg yeast, 200 pg HeLa + 50 pg and 240 pg HeLa +10 pg yeast were injected each. Peptides were separated at a throughput of 50 SPD. Data was recorded in DIA mode using optimal but not the same settings for the Orbitrap Astral MS and Orbitrap Exploris 480 MS and analyzed using DirectDIA+ in Spectronaut 18 at 1% FDR. Quantification was done on MS1 level. Dots within the Bland–Altman plots (bottom) represent proteins quantified based on at least 2 peptides with given $\log_2$ average PG abundance and $\log_2$-fold change of abundance across both proteome mixes. Density plots (right) depict the distribution of measured $\log_2$-fold changes and CV diagrams (top) show the local CV of 100 proteins quantified with a rolling window over the entire abundance range, n=3 technical replicates. For the Orbitrap Astral MS all quantified proteins (**a**, **d**, **g**) or only those proteins that were commonly quantified using the Orbitrap Exploris 480 MS (**b**, **e**, **h**) are shown. For the Orbitrap Exploris 480 MS, all quantified proteins are shown (**c**, **f**, **i**).

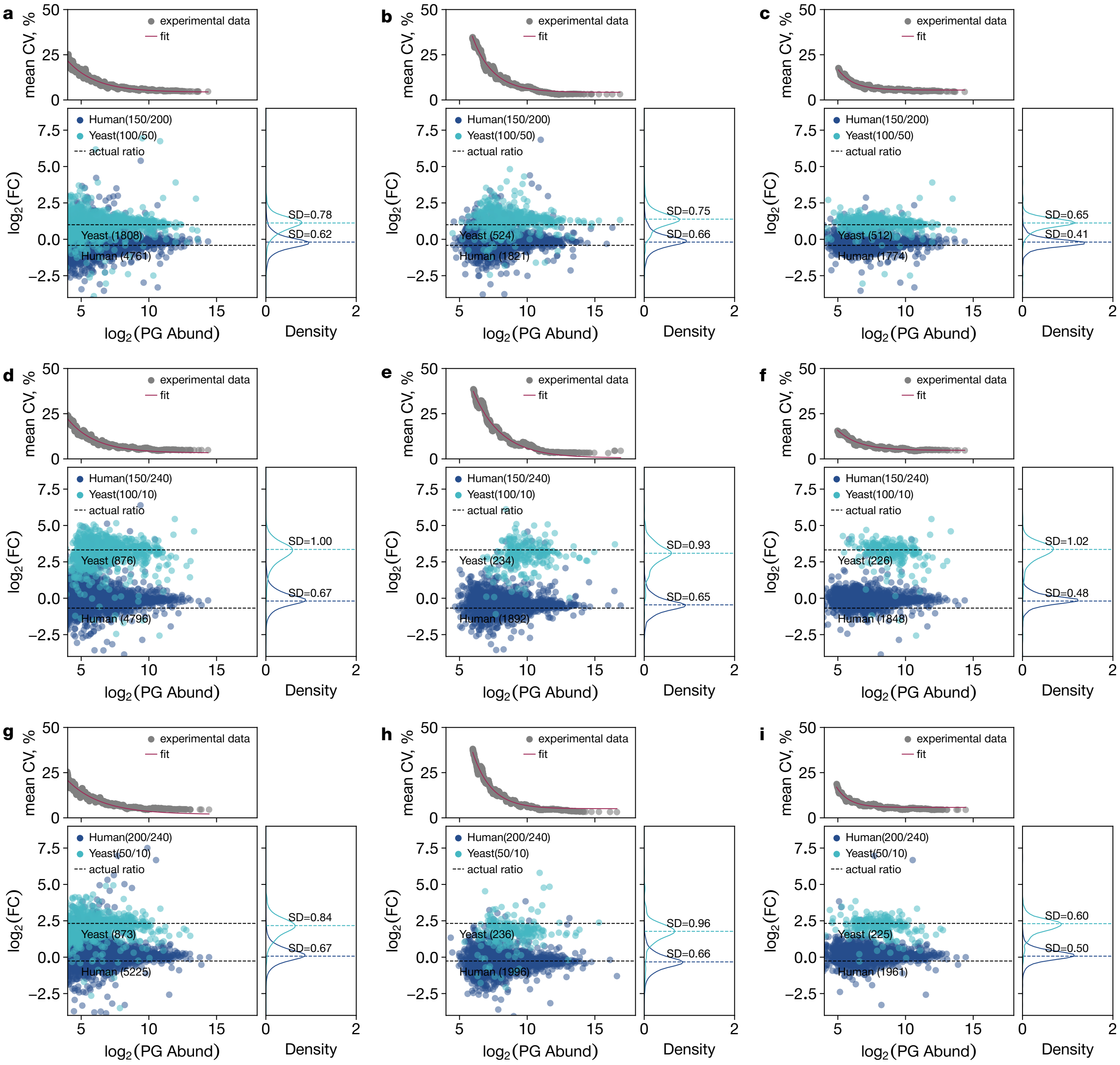


**Figure S7: Human–Yeast proteome mix to assess quantitative precision and accuracy of Orbitrap Astral and Orbitrap Exploris 480 mass spectrometers. MS2-based quantitation.** From diluted bulk digests 250 pg two-proteome mixes consisting of 150 pg HeLa + 100 pg yeast, 200 pg HeLa + 50 pg and 240 pg HeLa +10 pg yeast were injected each. Peptides were separated at a throughput of 50 SPD. Data was recorded in DIA mode using optimal but not the same settings for the Orbitrap Astral MS and Orbitrap Exploris 480 MS and analyzed using Direct DIA+ in Spectronaut 18 at 1% FDR. Quantification was done on MS2 level. Dots within the Bland–Altman plots (bottom) represent proteins quantified based on at least 1 peptide with given $\log_2$ average PG abundance and $\log_2$-fold change of abundance across both proteome mixes. Density plots (right) depict the distribution of measured $\log_2$-fold changes and CV diagrams (top) show the local CV of 100 proteins quantified with a rolling window over the entire abundance range, n=3 technical replicates. For the Orbitrap Astral MS all quantified proteins (**a**, **d**, **g**) or only those proteins that were commonly quantified using the Orbitrap Exploris 480 MS (**b**, **e**, **h**) are shown. For the Orbitrap Exploris 480 MS, all quantified proteins are shown (**c**, **f**, **i**).

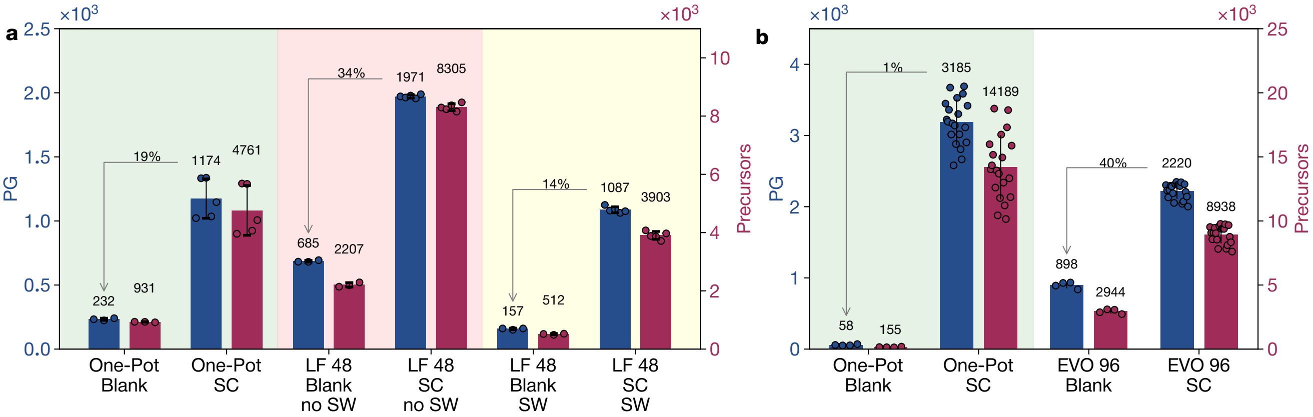


**Figure S8: Workflow benchmark. One-Pot 384-well in comparison with LF 48 and EVO 96 ProteoCHIPs.** Single cells were isolated, lysed and digested using the CellenONE robot either using the previously published One-Pot 384-well protocol, the LF 48 or the EVO 96 ProteoCHIP as indicated. Data was acquired on an Orbitrap Exploris 480 mass spectrometer at 50 SPD and data analysis was performed in DirectDIA+ mode using Spectronaut 18. Dots represent identified precursors or protein groups from individual replicates, bars show mean values and error bars indicate standard deviations. A. Comparison of One-Pot 384-well (Armadillo) to the (reused) LF 48 CHIP with or without an additional stringent washing (SW) to reduce background contamination. Single HeLa cells of 18-25 µm. **b**: Comparison of One-Pot 384-well (Armadillo) to the (reused) EVO 96 CHIP protocol. Single A549 cells of 20-30 µm.

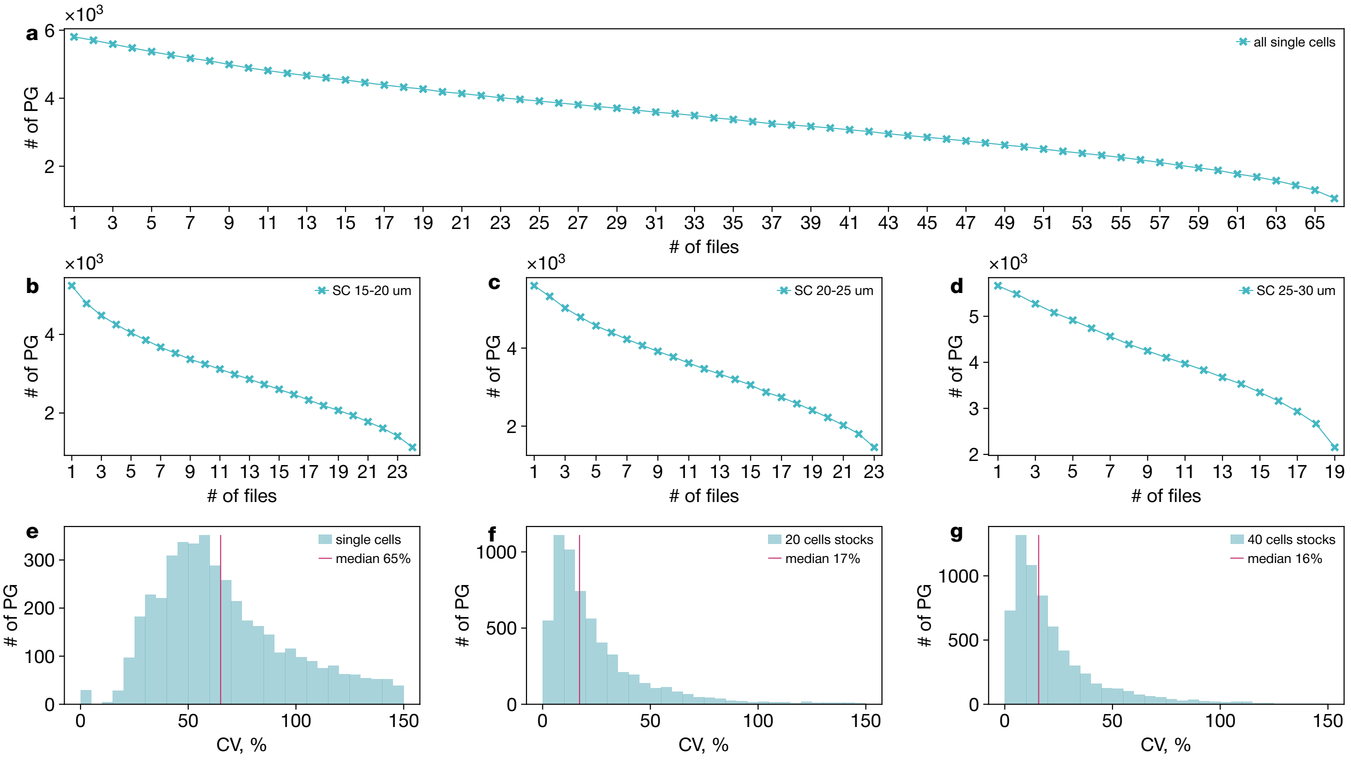


**Figure S9: Data completeness and Coefficients of Variation in A549 dataset.** Data completeness at protein group level of all 66 single-cell runs (**a**). **b**, **c**, **d**: data completeness in subgroups of A549 cells depending on the diameter of analyzed cell: 15-20 µm, 20-25 µm and 25-30 µm. DirectDIA+ search results of Spectronaut 18 were used for the figure. CV for single-cell runs (**e**), 20-cell (**f**) and 40-cell (**g**) stock runs of A549 cell line dataset. The blue line indicates the median of the distribution. Protein abundance quantitation is based on MS1 intensities. DirectDIA+ search results of Spectronaut 18 were used for the figure.

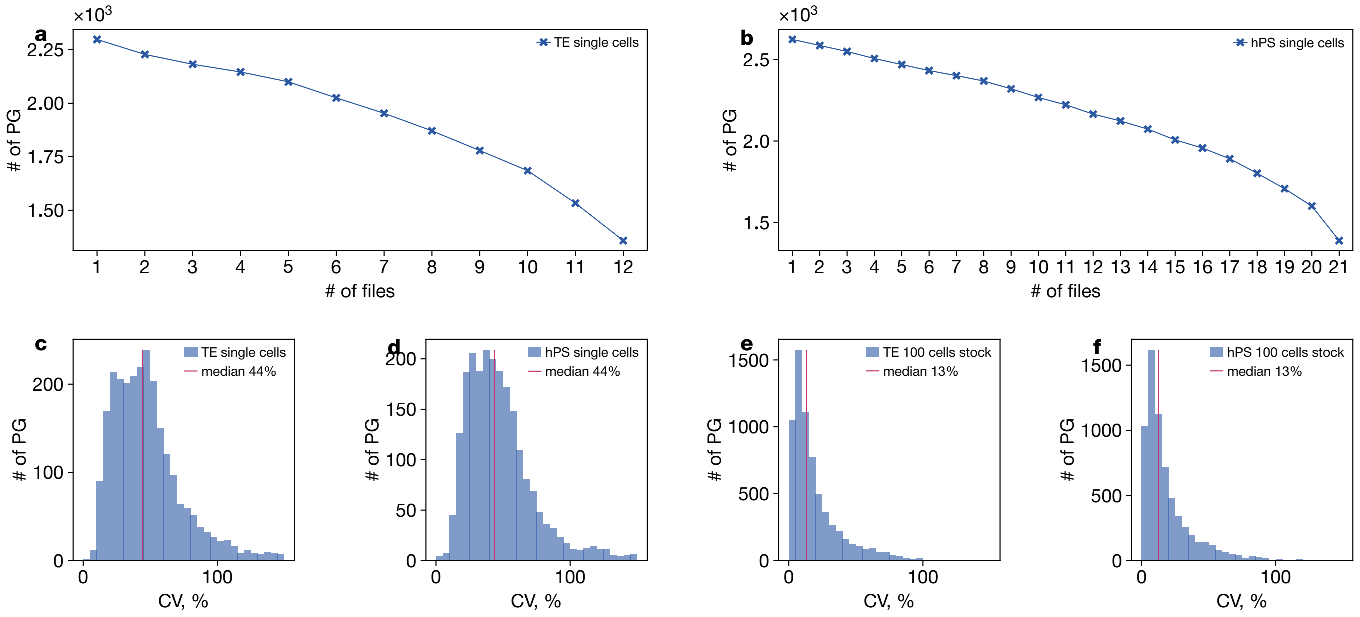


**Figure S10: Data completeness and Coefficients of Variation in TE/hPSC dataset.** Data completeness at protein group level of all single-cell runs of TE (**a**) and hPSC (**b**). DirectDIA+ search results of Spectronaut 18 were used for the figure. CV for TE and hPSC dataset of **c**: TE single-cell runs; **d**: hPSC single-cell runs; **e**: TE 100-cell runs; **f**: hPSC 100-cell runs. The red line indicates the median of the distribution. Protein abundance quantitation is based on MS1 intensities. DirectDIA+ search results of Spectronaut 18 were used for the figure.

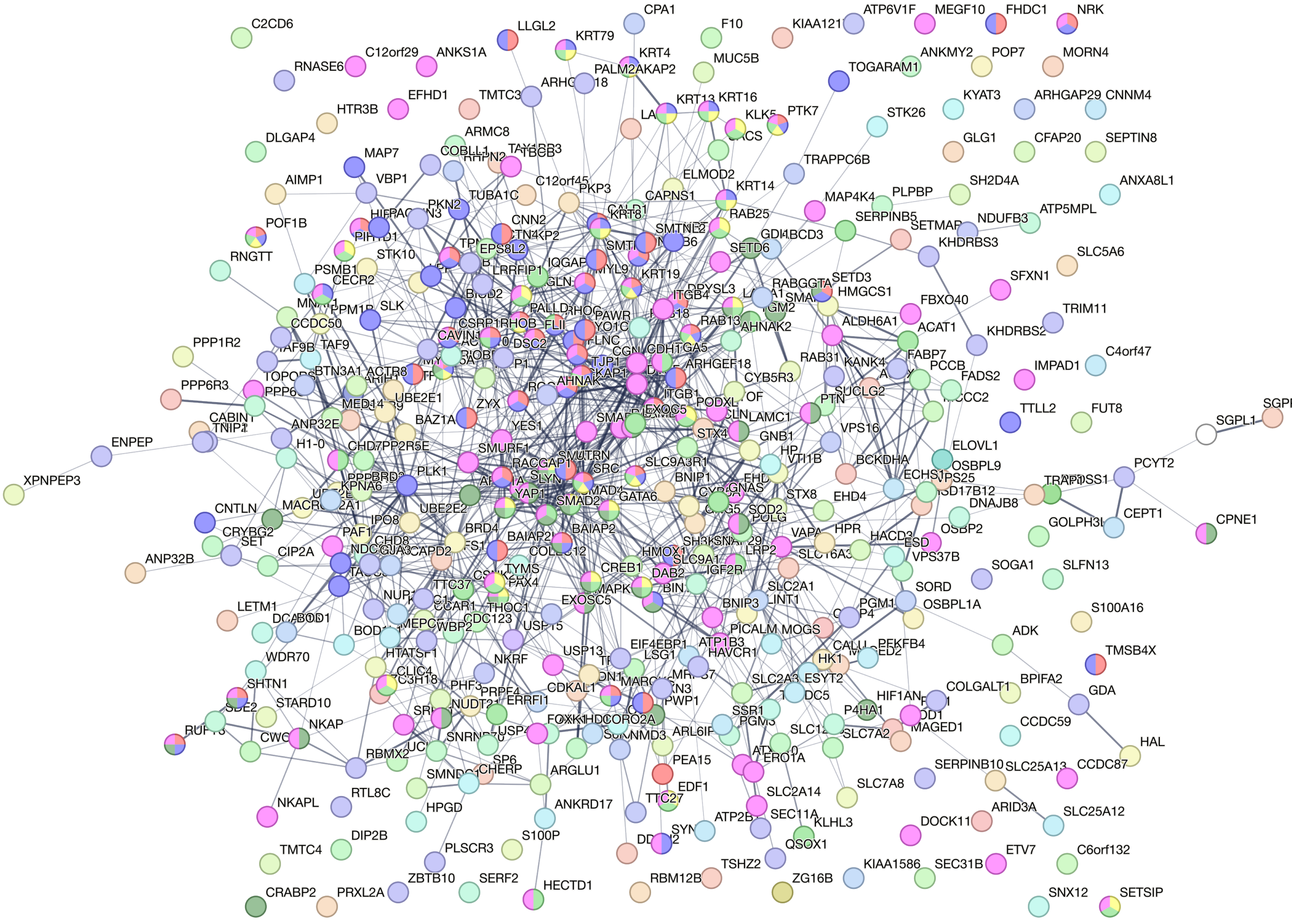

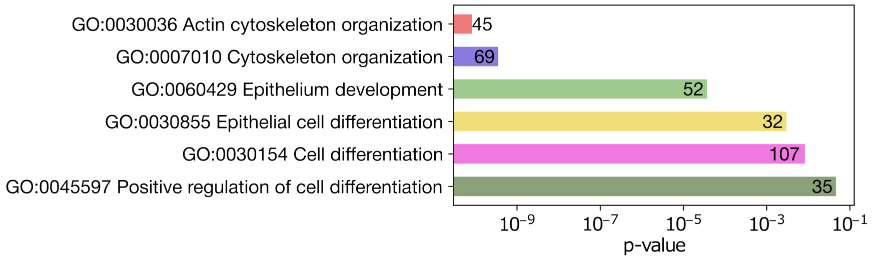


**Figure S11: GO analysis of upregulated proteins in TE-like cells with highlighted selected biological pathways (single-cell data).** GO analysis was performed using String-db.org. At the bottom: for highlighted biological pathways $p$-values corrected for multiple testing within each category using the Benjamini–Hochberg procedure are shown. The number next to each bar corresponds to differentially expressed proteins found in this study for a pathway.

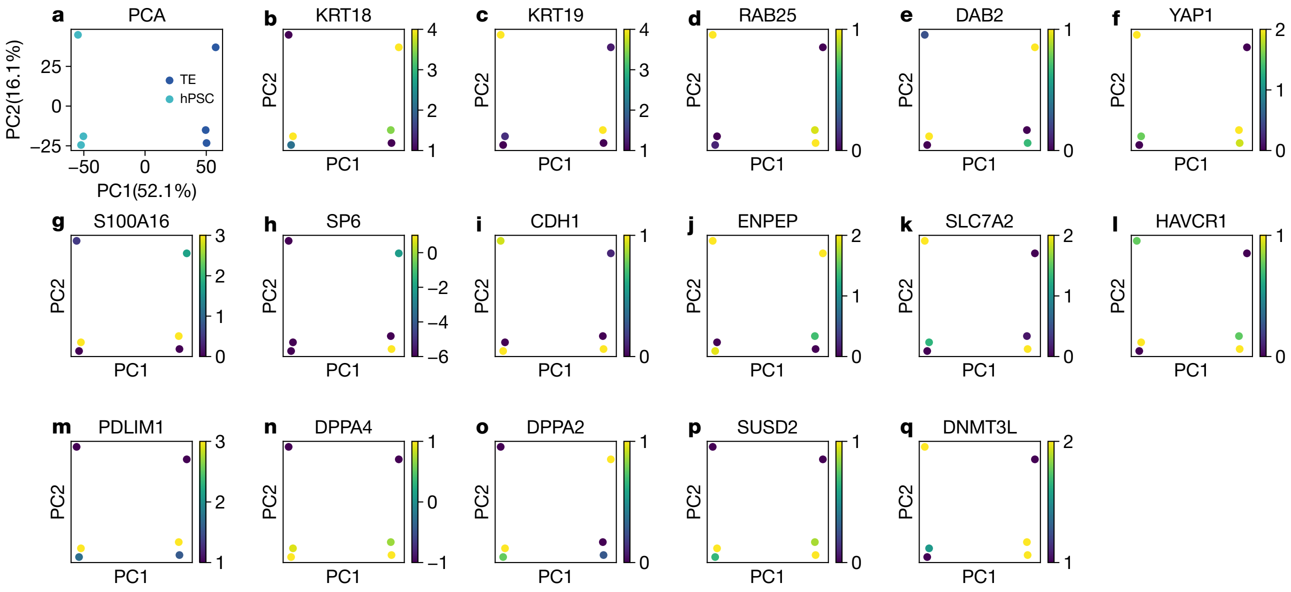


**Figure S12: Distribution of protein abundance in TE vs hPSC cells on a PCA plot (100-cells data).** PCA analysis of 100-cell runs of TE and hPSC. was based on protein quantities with each dot representing a 100-cell run and colors reflecting the cell type based on their fluorescent marker proteins. Distribution of protein abundance in TE vs hPSC cells on a PCA plot (100-cell data).

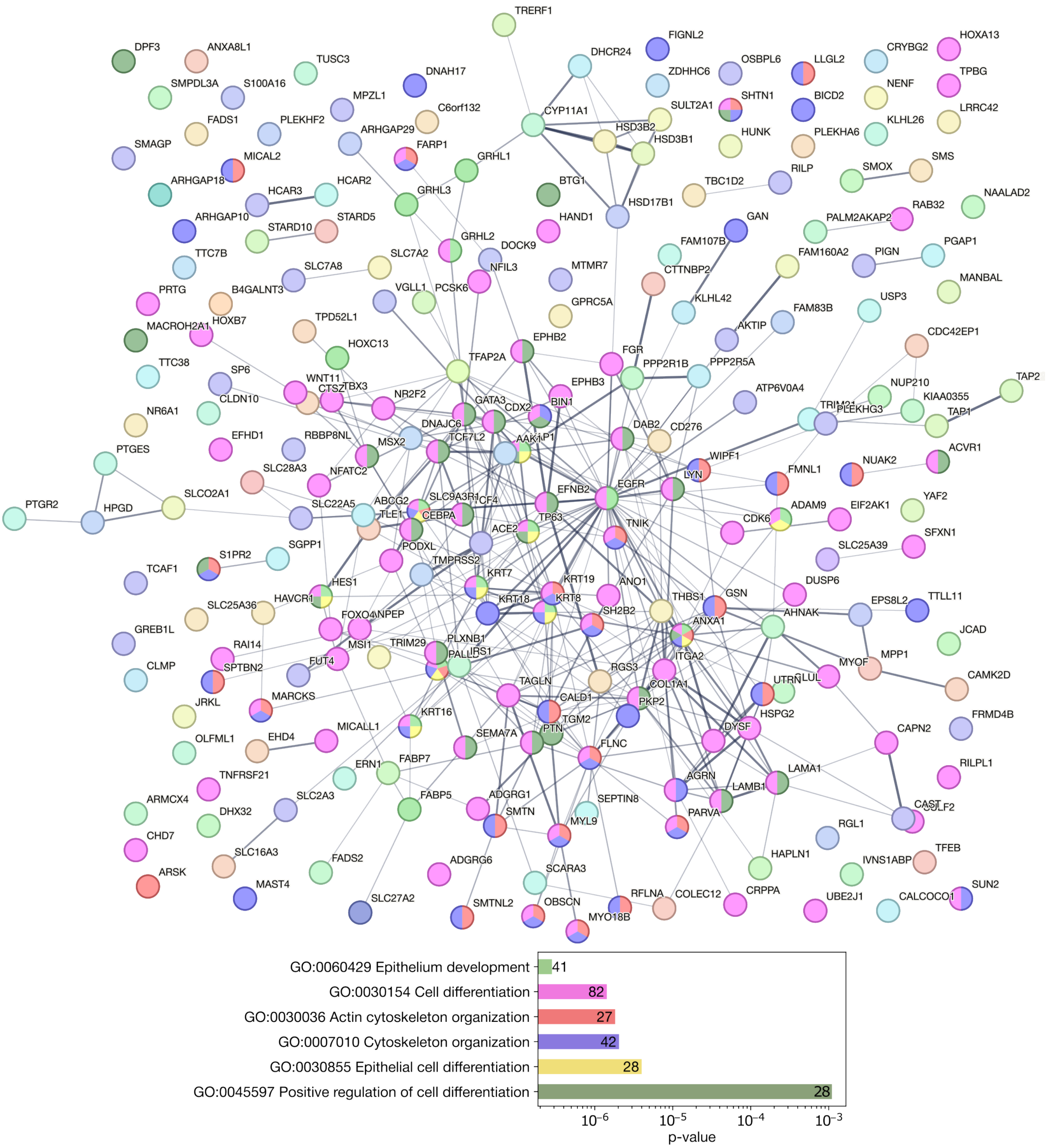


**Figure S13: GO analysis of upregulated proteins in TE cells with highlighted selected biological pathways (100-cells data).** GO analysis was performed using String-db.org. At the bottom: for highlighted biological pathways $p$-values corrected for multiple testing within each category using the Benjamini–Hochberg procedure are shown. The number next to each bar corresponds to differentially expressed proteins found in this study for a pathway.

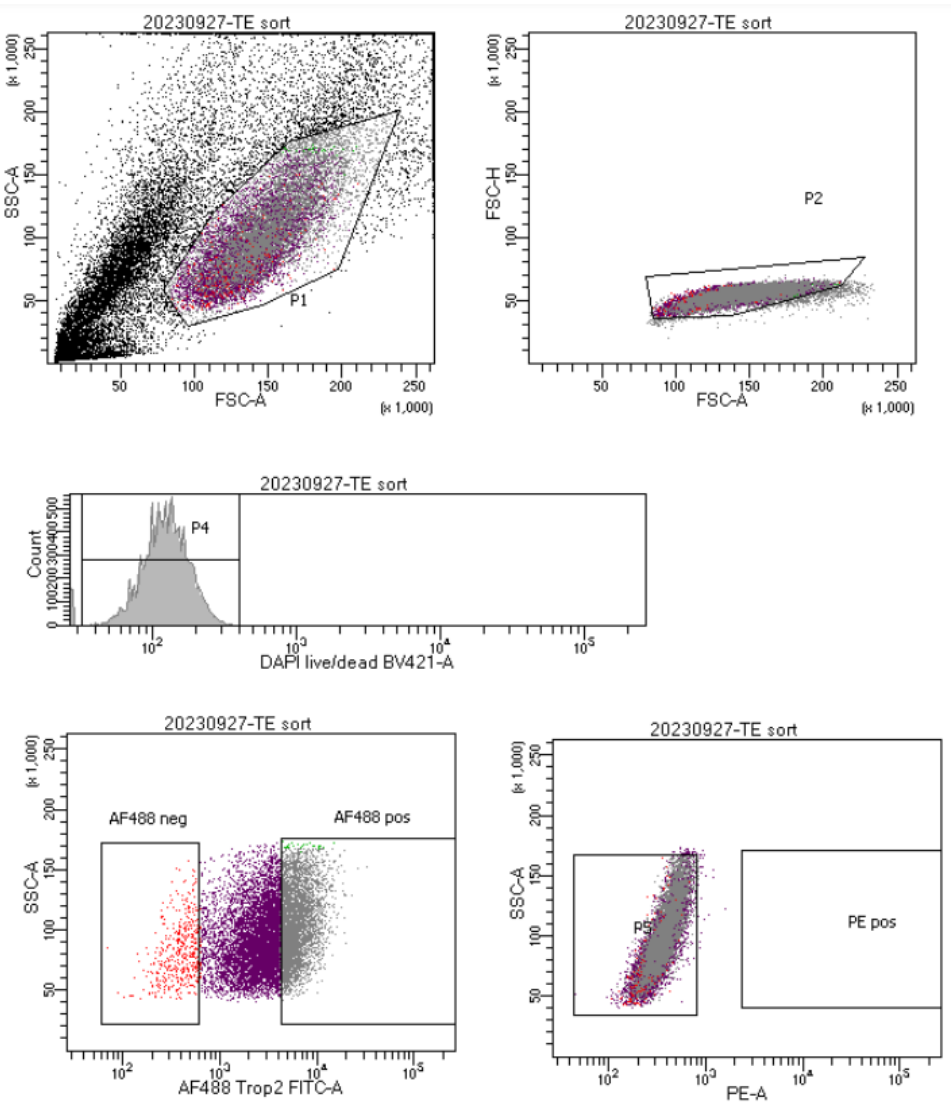


**Figure S14: Gating strategy for the isolation of individual TE cells using flow cytometry.** Initial gating was performed to exclude debris based on forward scatter (FSC) and side scatter (SSC) properties. Single cells were identified by gating on FSC-A vs. FSC-H. Dead cells were excluded using DAPI staining. TROP2 (AF 488 positive) was used as TE marker.

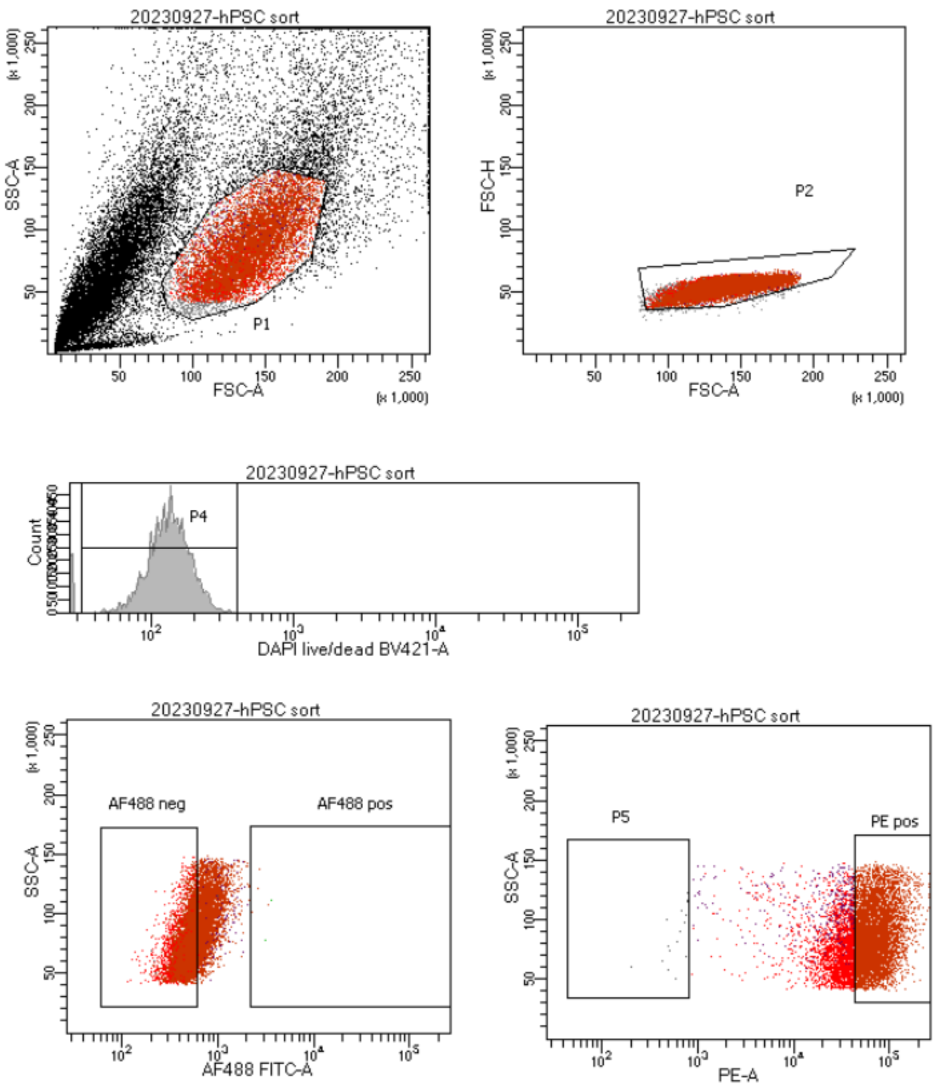


**Figure S15: Gating strategy for the isolation of individual hPSCs using flow cytometry.** Initial gating was performed to exclude debris based on FSC and SSC properties. Single cells were identified by gating on FSC-A vs. FSC-H. Dead cells were excluded using DAPI staining. SUSD2 (PE pos) was used as hPSC marker.

# References


1. Matzinger, M., Müller, E., Dürnberger, G., Pichler, P. & Mechtler, K. Robust and Easy-to-Use One-Pot Workflow for Label-Free Single-Cell Proteomics. *Analytical chemistry* **95,** 4435–4445 (2023).

2. Krisp, C. *et al.* Combining cellenONE® and timsTOF SCP for easy and sensitive, end-to-end label-free single cell proteomics workflows. (2022).

3. Krisp, C. *et al.* Pushing the boundaries for robust and high-throughput single cell analysis. *Technote-Evosep* (2022).
