# Supplementary material for: Challenging the Astral mass analyzer to quantify up to 5,300 proteins per single cell at unseen accuracy to uncover cellular heterogeneity
Source: Nat Methods. 2025 Jan 16;22(3):510–9. doi: 10.1038/s41592-024-02559-1 (PMC11903296; doi:10.1038/s41592-024-02559-1)
Supplement: Supplementary file 2 — Reporting Summary [file 41592_2024_2559_MOESM2_ESM.pdf]

nature portfolio

Corresponding author(s): Julia Bubis, Karl Mechtler, Manuel Matzinger

Last updated by author(s): Sep 25, 2024

Reporting Summary

Nature Portfolio wishes to improve the reproducibility of the work that we publish. This form provides structure for consistency and transparency in reporting. For further information on Nature Portfolio policies, see our Editorial Policies and the Editorial Policy Checklist.

Statistics

For all statistical analyses, confirm that the following items are present in the figure legend, table legend, main text, or Methods section.

n/a	Confirmed	
☐	☒	The exact sample size (n) for each experimental group/condition, given as a discrete number and unit of measurement
☐	☒	A statement on whether measurements were taken from distinct samples or whether the same sample was measured repeatedly
☐	☒	The statistical test(s) used AND whether they are one- or two-sided<br>*Only common tests should be described solely by name; describe more complex techniques in the Methods section.*
☐	☒	A description of all covariates tested
☐	☒	A description of any assumptions or corrections, such as tests of normality and adjustment for multiple comparisons
☐	☒	A full description of the statistical parameters including central tendency (e.g. means) or other basic estimates (e.g. regression coefficient) AND variation (e.g. standard deviation) or associated estimates of uncertainty (e.g. confidence intervals)
☐	☒	For null hypothesis testing, the test statistic (e.g. F, t, r) with confidence intervals, effect sizes, degrees of freedom and P value noted<br>*Give P values as exact values whenever suitable.*
☒	☐	For Bayesian analysis, information on the choice of priors and Markov chain Monte Carlo settings
☐	☒	For hierarchical and complex designs, identification of the appropriate level for tests and full reporting of outcomes
☒	☐	Estimates of effect sizes (e.g. Cohen's d, Pearson's r), indicating how they were calculated

Our web collection on statistics for biologists contains articles on many of the points above.

Software and code

Policy information about availability of computer code

Data collection	Mass spectrometry data was acquired using the Orbitrap Astral MS or Orbitrap Exploris 480 MS, using Thermo Tune software (version: 0.4 or higher). Cell sizes were recorded on the cellenONE using its control software (v2.0-1143).
Data analysis	Proteomics data was analysed using Spectronaut (v. 18.6.231227.55695). For FDR checks a decoy ("shuffled target") database was generated using the Pyteomics (v. 4.6.2) Python package PCA and UMAP clustering were done using the sklearn (v. 1.2.0) and umap (v. 0.5.6) Python packages. t-test and FDR correction for multiple testing were performed using scipy (v. 1.11.4) and statsmodel (v. 0.14.1). Heatmap clustering was done using seaborn (v. 0.12.2) Python package. Python packages, respectively. Gene Ontology analysis was performed using string - db.org (v. 12.0).

For manuscripts utilizing custom algorithms or software that are central to the research but not yet described in published literature, software must be made available to editors and reviewers. We strongly encourage code deposition in a community repository (e.g. GitHub). See the Nature Portfolio guidelines for submitting code & software for further information.

Data

Policy information about availability of data

All manuscripts must include a data availability statement. This statement should provide the following information, where applicable:

- Accession codes, unique identifiers, or web links for publicly available datasets
- A description of any restrictions on data availability
- For clinical datasets or third party data, please ensure that the statement adheres to our policy

The mass spectrometry proteomics data along with FASTA files and result files have been deposited to the ProteomeXchange Consortium via the PRIDE partner repository with the dataset identifier PXD049412. Data analysis of Spectronaut search results and figure plotting are available on GitHub via link: https://github.com/SimpleNumber/SPC_on_AstralMS

Human research participants

Policy information about studies involving human research participants and Sex and Gender in Research.

Reporting on sex and gender	n.a.
Population characteristics	n.a.
Recruitment	n.a.
Ethics oversight	n.a.

Note that full information on the approval of the study protocol must also be provided in the manuscript.

Field-specific reporting

Please select the one below that is the best fit for your research. If you are not sure, read the appropriate sections before making your selection.

☒ Life sciences ☐ Behavioural & social sciences ☐ Ecological, evolutionary & environmental sciences

For a reference copy of the document with all sections, see nature.com/documents/nr-reporting-summary-flat.pdf

Life sciences study design

All studies must disclose on these points even when the disclosure is negative.

Sample size	To assess technical variability of the mass spectrometry measurements, each experiment was performed using at least three injection replicates. No sample size calculation was performed. As we saw a very low variance between these technical replicates aiming to check on the reproducibility of the instrument workflows themselves, we decided that no further replicates were needed.<br>For single-cell experiments 20 A549 and H460 cells where compared, for cell cycle investigations 66 A549 cells were analyzed and a total of 33 individual TE/hPSC cells was analyzed. For library generation and pseudo bulk analyses, 20, 40 or 100 cells were used as indicated in the main text. At least 3 blank controls were included in every single-cell study.<br>No sample size calculation was performed, but heterogeneity between cell types/cells was clearly visible with the number of measured replicates, indicating it is sufficient. We assume, even more cell replicates are always better, but are limited in measurement time.
Data exclusions	Single cells were excluded from downstream analysis if the number of identified proteins was less that 3 times number of proteins in the blanks.
Replication	All experiments were performed using 3 replicates of which all were successful
Randomization	For technical/methodological benchmarks no randomization was applied. Randomization/control of covariates is not applicable here as all 3 technical replicates are injected from the same vial and are hence identical. For single cell measurements, cells are selected randomly based on the design of the sample prep and filtered only for their size and circularity to exclude debris, multiple cells/well or dead cells.
Blinding	No blinding was performed or relevant to this work. A comprehensive overview of all LC-MS methods and sample preperation methods benchmarked in this study was needed to select the best perfoming option. Knowledge of cell size and type compared in this study was required to check if they were properly separated in our heterogenity studies (PCA, UMAP).<br>Technical replicates were used to assess precision and accuracy, and randomization was considered not relevant as we focused on improving and validating the capabilities of our MS but did not compare any sample treatments. Also no clinical sample were used in this study.

Reporting for specific materials, systems and methods

We require information from authors about some types of materials, experimental systems and methods used in many studies. Here, indicate whether each material, system or method listed is relevant to your study. If you are not sure if a list item applies to your research, read the appropriate section before selecting a response.

Materials & experimental systems

n/a	Involved in the study
☐	☒ Antibodies
☐	☒ Eukaryotic cell lines
☒	☐ Palaeontology and archaeology
☒	☐ Animals and other organisms
☒	☐ Clinical data
☒	☐ Dual use research of concern

Methods

n/a	Involved in the study
☒	☐ ChIP-seq
☐	☒ Flow cytometry
☒	☐ MRI-based neuroimaging

Antibodies

Antibodies used	Human TROP-2 Alexa Fluor 488 conjugated antibody, R and D system FAB650G-100UG,Lot:1646351<br>Anti-SUSD2-PE Miltenyi Biotec Cat#: 130-117-682; Clone : W5C5, LOT: 5201104670
Validation	Detects human TROP-2 in direct ELISAs and Western blots. In direct ELISAs, no cross-reactivity with recombinant human (rh) VCAM1 or rhICAM1 is observed.<br>Validation statements available from manufacturers:<br>anti-TROP2 (https://www.rndsystems.com/products/human-trop-2-antibody-77220_mab650)<br>anti-SUSD2 (https://www.miltenyibiotec.com/AT-en/products/susd2-antibody-anti-human-w5c5.html#gref)

Eukaryotic cell lines

Policy information about cell lines and Sex and Gender in Research

Cell line source(s)	A549 and H460 cells were provided by the laboratory of Josef Penninger, their original commercial source is ATCC.<br>Human embryonic stem cells (Wicell line H9) reset to naive state were provided by the Laboratory of Yasuhiro Takashima
Authentication	H9 was included in single cell sequencing analysis to authenticate their identity in previous studies of Nicolas Rivron. We comfirm naïve state of the cells by assessing the expression of the naïve PSC marker SUSD2 through staining. Additionally, we routinely thaw a fresh stock vial to minimize the risk of cross-contamination with unintended cell lines and to prevent the accumulation of DNA mutations
Mycoplasma contamination	Cells were routinely tested for mycoplasma contamination. No contamination was detected
Commonly misidentified lines (See ICLAC register)	none

Flow Cytometry

Plots

Confirm that:

☒ The axis labels state the marker and fluorochrome used (e.g. CD4-FITC).

☒ The axis scales are clearly visible. Include numbers along axes only for bottom left plot of group (a 'group' is an analysis of identical markers).

☒ All plots are contour plots with outliers or pseudocolor plots.

☒ A numerical value for number of cells or percentage (with statistics) is provided.

Methodology

Sample preparation	TE-like cells and naïve PSCs were dissociated using Accutase at 37°C for 10 minutes and 5 minutes, respectively. Gentle mechanical dissociation was performed using a pipette. Cells were then stained with antibodies against TROP2 and SUSD2, respectively.
Instrument	FACS Aria III (BD)
Software	DiVa version on the F02 is 9.0.1

Cell population abundance	Abundance of the cell populations of interest was determined by the appropriate negative control and the purity of sorted population was assessed by the post sort analysis.
Gating strategy	FSC-A/SSC-A and SSC-H/SSC-W gates were applied to remove debris, and non-single cell aggregates respectively. Dead cells were excluded by using DAPI signal.

☒ Tick this box to confirm that a figure exemplifying the gating strategy is provided in the Supplementary Information.
